# Adolescence As Risk Factor for Adverse Pregnancy Outcome in Central Africa – A Cross-Sectional Study

**DOI:** 10.1371/journal.pone.0014367

**Published:** 2010-12-20

**Authors:** Florian Kurth, Sabine Bélard, Ghyslain Mombo-Ngoma, Katharina Schuster, Ayola A. Adegnika, Marielle K. Bouyou-Akotet, Peter G. Kremsner, Michael Ramharter

**Affiliations:** 1 Medical Research Unit, Albert Schweitzer Hospital, Lambaréné, Gabon; 2 Department of Neonatology and Paediatric Intensive Care, University Hospital Carl Gustav Carus, Dresden, Germany; 3 Institute of Tropical Medicine, University of Tübingen, Tübingen, Germany; 4 Department of Paediatrics and Adolescent Medicine, University Freiburg, Freiburg, Germany; 5 Département de Parasitologie, Mycologie, Médecine Tropicale, Université des Sciences de la Santé, Libreville, Gabon; 6 Department of Parasitology, Leiden University Medical Center, Leiden, The Netherlands; 7 Division of Infectious Diseases and Tropical Medicine, Department of Medicine I, Medical University of Vienna, Vienna, Austria; University of Cape Town, South Africa

## Abstract

**Background:**

Sub-Saharan Africa has the highest rates of maternal and neonatal mortality worldwide. Young maternal age at delivery has been proposed as risk factor for adverse pregnancy outcome, yet there is insufficient data from Sub-Saharan Africa. The present study aimed to investigate the influence of maternal adolescence on pregnancy outcomes in the Central African country Gabon.

**Methodology and Principal Findings:**

Data on maternal age, parity, birth weight, gestational age, maternal *Plasmodium falciparum* infection, use of bednets, and intake of intermittent preventive treatment of malaria in pregnancy were collected in a cross-sectional survey in 775 women giving birth in three mother-child health centers in Gabon. Adolescent women (≤16 years of age) had a significantly increased risk to deliver a baby with low birth weight in univariable analysis (22.8%, 13/57, vs. 9.3%, 67/718, OR: 2.9, 95% CI: 1.5–5.6) and young maternal age showed a statistically significant association with the risk for low birth weight in multivariable regression analysis after correction for established risk factors (OR: 2.7; 95% CI: 1.1–6.5). In further analysis adolescent women were shown to attend significantly less antenatal care visits than adult mothers (3.3±1.9 versus 4.4±1.9 mean visits, p<0.01, n = 356) and this difference accounted at least for part of the excess risk for low birth weight in adolescents.

**Conclusion:**

Our data demonstrate the importance of adolescent age as risk factor for adverse pregnancy outcome. Antenatal care programs specifically tailored for the needs of adolescents may be necessary to improve the frequency of antenatal care visits and pregnancy outcomes in this risk group in Central Africa.

## Introduction

Sub-Saharan Africa accounts for half of the world's burden of maternal, newborn and child death with over 13,000 mothers, newborns and children dying every day [1], [2]. Improvements in maternal, newborn and child health are therefore among the most pressing health challenges in Africa. To foster the international commitment, the UN-Millennium Development Goals defined the aim to reduce maternal mortality by three quarters and the mortality of children below the age of 5 years by two thirds until 2015 [3].

In Africa high rates of maternal and neonatal morbidity and mortality are a consequence of multiple causes including endemic infectious diseases (malaria, HIV/AIDS, tuberculosis), malnutrition and micronutrient deficiencies, child-birth complications, newborn illness and inadequate antenatal and perinatal care due to financial and logistic constraints in these resource poor regions [1], [4], [5]. Public health interventions such as intermittent preventive treatment of malaria, vitamin supplementation, insecticide treated bednets, skilled birth attendance and increasing the frequency and quality of antenatal care are therefore the cornerstone of current strategies to reduce pregnancy associated adverse health outcomes [6]–[9]. The identification of additional - yet underappreciated - preventable risk factors for adverse pregnancy outcome is necessary to further strengthen current efforts to reduce maternal and neonatal mortality.

Adolescent pregnancy is defined as gestation of women before having reached the full somatic development [10].The percentage of childbearing adolescent women is regionally highly variable depending on cultural, religious, political, economic and other factors [11]. Early pregnancy has been discussed as an independent risk factor for adverse pregnancy outcome. Large epidemiologic studies on this topic were however largely reported from high or medium income countries showing conflicting results [12], [13]. Most importantly to date there is a lack of epidemiologic evidence from Sub-Saharan Africa – a region with multiple risk factors for adverse pregnancy outcomes and regionally highly variable rates of adolescent pregnancies [14]–[16]. The aim of our study was therefore to investigate the potential influence of adolescent pregnancy on maternal and neonatal health outcomes in Central Africa.

## Methods

### Ethics Statement

The study protocol was approved by the Ethics Committee of the International Foundation for the Albert Schweitzer Hospital in Lambaréné. Written informed consent was sought from the mother and the guardian accompanying the patient to the hospital.

### Study design, study site, participants

From May 2005 to September 2006 a cross-sectional study in women delivering at the obstetric departments of local hospitals in the cities of Libreville (Centre Hospitalier de Libreville) and Lambaréné (Hôpital Régional de Lambaréné and Albert Schweitzer Hospital) was conducted in the Central African country Gabon. The region is characterized by perennial high transmission of *Plasmodium falciparum* malaria [17], [18]. Intermittent preventive treatment in pregnancy with sulfadoxine-pyrimethamine and the use of insecticide treated bednets have been adopted as national policy for the prevention of malaria in pregnancy since 2005. HIV prevalence among pregnant women is estimated to be between 5% and 10%. Libreville – the capital of Gabon –is a typical Central African city whereas Lambaréné – situated approximately 300 km inland – is characterized by a semi-urban environment and is located in a predominantly rural province.

Women in labour attending the hospital for delivery were eligible for participation in this survey. Information on maternal age, last date of menstruation, parity, number of antenatal consultations, use of bednets during pregnancy and intake of sulfadoxine-pyrimethamine for intermittent preventive treatment of malaria were collected in structured interviews and from mother-child health booklets. A thick blood smear for the diagnosis of malaria was performed as well as maternal haemoglobin measurements (CellDyn 3000, Abbott Laboratories, Santa Clara, CA).

All newborns were weighed immediately after birth and birth outcome was evaluated by the midwife in charge of the delivering women. Only women giving birth to live singletons were considered for analysis. Data were captured on paper record forms and reviewed manually before analysis.

### Outcome variables, definitions and statistical analysis

The aim of the statistical analysis was to evaluate differences in pregnancy characteristics and birth outcomes of adolescent versus adult women. Adolescent status was defined as an age of or below 16 years at delivery. The number of antenatal consultations, use of bednet during pregnancy, intake of sulfadoxine-pyrimethamine for intermittent preventive treatment of malaria, maternal haemoglobin at delivery, and peripheral parasitaemia at delivery were specified as main pregnancy characteristics. Continuous and categorical measures of birth weight and gestational age at delivery were defined as main birth outcome variables. Gestational age was calculated based on the last date of menses as reported by the pregnant women. Birth weight below 2500g was defined as low birth weight and deliveries before 37 weeks of gestation were classified as preterm deliveries. Univariable analysis of epidemiological data for the two groups (adolescent and adult pregnant women) was performed by Student's t-test at a two-sided significance level of α = 0.05 for continuous variables and odds ratios for categorical variables.

Since parity is a known risk factor for low birth weight an analysis of nulliparous adolescent and adult mothers was performed to correct for differences in parity between the two groups. In addition a separate analysis of differences in birth weight was carried out in the subset of term-newborns in order to account for the strong influence of preterm delivery on the occurrence of low birth weight. Finally, a comparative graphical analysis of birth weight for each completed week of gestation was performed in the subset of term-newborns to appreciate potential differences of growth retardation in adolescent and adult mothers over time.

To further analyse the impact of adolescent pregnancy on low birth weight multivariable logistic regression analysis was performed. For this purpose statistical significance of variables associated with low birth weight was computed. Known risk factors for low birth weight and variables showing a statistically significant association with low birth weight in our study population were included as dichotomized variables in the logistic regression analysis. Data are displayed as mean (±standard deviation) or median (±interquartile range). Statistical analysis was performed using JMP (JMP 7.0, SAS Institute Inc., NC, USA).

## Results

### Participants' characteristics

A total of 775 pregnant women delivering at the Centre Hospitalier de Libreville (n = 591) or at one of the two hospitals in Lambaréné (n = 184) were included in this epidemiological survey. Pregnant women participating in the study had a mean age of 24 years. The age ranged from 13 to 45 years. Parity ranged from 0 to 15 and 275 (37%) women had not given birth before. The number of pregnant women with an age of or below 15, 16, 17, and 18 years was 29 (4%), 57 (7%), 90 (12%) and 142 (18%), respectively. Intake of at least one or two doses of intermittent preventive treatment of malaria in pregnancy was recorded in 607 (86%) and 414 (60%) women, respectively. Bednet use was reported by 329 (50%) mothers.

Peripheral parasitaemia at delivery was detected in 13 (2%) women. Newborns had a mean birth weight of 3077±500 g and 80 (10%) children were classified as presenting with low birth weight. Reliable information on last date of menses could be obtained in 661 cases and preterm birth was recorded in 126 (19%) children. Out of the 69 children with low birth weight for whom information on gestational age was available, 38 (55%) were classified as preterm newborns. Information on the number of antenatal care visits was obtained in 356 cases and a median (IQR) of 4 [3]–[6] antenatal care visits was recorded for mothers in this survey.

### Univariable analysis of adolescent pregnancies compared to adult pregnancies

The proportion of infants with low birth weight was 23% (13/57) in adolescent women compared to 9% (67/718) in women older than 16 years (OR: 2.9, 95%CI: 1.5–5.6). Accordingly, children born to adolescents had an average birth weight which was 304g lower than those born to adults (2795±514g versus 3099±493g, p<0.0001, n = 775). The mean gestational age at delivery was comparable between both groups (38.5±2.5 weeks vs. 38.8±2.7 weeks, p = 0.4, n = 661, for adolescent and adult women, respectively) and there was no statistically significant difference in the occurrence of preterm birth (13% vs. 19%, OR: 1.3 95%CI: 0.6–2.6, n = 661). The prevalence of a peripheral blood smear positive for *P. falciparum* was significantly higher in adolescent mothers than in mothers older than 16 years (7% (4/57) vs. 1% (9/718), OR 5.9, 95%CI: 1.7–19.9, n = 775).

Since adolescent women are more likely to give birth to a first child and primiparity is an established risk factor for adverse birth outcome the same statistical analysis was performed for nulliparous women only (n = 275). In this analysis differences between the two groups were statistically significant for mean birth weight (2823±471g vs. 2971±443g, p<0.01, n = 275) and the prevalence of a peripheral blood smear positive for *P. falciparum* (7% (4/56) vs. 2% (4/219), OR 4.1, 95%CI: 1.1–17.1). Table 1 depicts a summary of birth outcomes in adolescent mothers compared to mothers older than 16.

**Table 1:** Univariable analysis of adolescent compared to adult women.

Assessments (Number of Women)	Adolescent (≤16 years)	Adult (>16 years)	
**All mothers:**			
Birth weight (775)	2795±514g	3099±493g	p<0.0001
LBW (775)	22.8% (13/57)	9.3% (67/718)	OR 2.9, 95%CI: 1.5–5.6
Gestational age (661)	38.5±2.6wk	38.7±2.7wk	p = 0.46
Prematurity (661)	22.9% (11/48)	18.8% (115/613)	OR 1.3, 95%CI: 0.6–2.6
Peripheral parasitaemia (775)	7.0% (4/57)	1.2% (9/718)	OR 5.9, 95%CI: 1.7–19.9
No. of consultations (356)	3.3±1.9	4.4±1.9	p<0.01
Consultations < = 3 (356)	58.3% (14/24)	33.4% (111/332)	OR 2.8, 95%CI: 1.2–6.5
Use of Bednet (646)	45.1% (23/51)	51.4%(306/595)	OR 0.8, 95%CI: 0.4–1.4
Intake of 1 dose IPTp (705)	86.5% (45/52)	86.1% (562/653)	OR 1.0, 95%CI: 0.5–2.4
Intake of 2 doses IPTp (691)	55.8% (29/52)	60.3% (385/639)	OR 0.8, 95%CI: 0.5–1.5
Haemoglobin at delivery (342)	6.4±0.9 mmol/l	6.7±0.9 mmol/l	p = 0.71
**Primiparous women**			
Birth weight (275)	2823±471g	2971±443g	P<0.01
LBW (275)	21.4%(12/56)	12.3%(27/219)	OR 1.9, 95%CI: 0.9–4.1
Gestational Age	38.6±2.5wk	38.7±2.8wk	p = 0.83
Preterm Delivery (236)	21.3% (10/47)	19.1% (36/189)	OR 1.1, 95%CI: 0.5–2.5
Peripheral parasitaemia (275)	7.1% (4/56)	1.8% (4/215)	OR 4.1, 95%CI: 1.1–17.1
No. of consultations (133)	3.3±1.9	4.5±1.9	p<0.01
Consultations < = 3 (133)	58.37% (14/24)	37.6% (41/109)	OR 2.3, 95%CI: 0.9–5.7
Use of Bednet (236)	44.0% (22/50)	44.1% (82/104)	OR 1.0, 95%CI: 0.5–1.9
Intake of 1 dose IPTp (257)	88.3% (45/51)	84.9% (175/206)	OR 1.3, 95%CI: 0.5–3.3
Intake of 2 doses IPTp (524)	56.9% (29/51)	62.6% (127/203)	OR 0.8, 95%CI: 0.4–1.5
Haemoglobin at delivery (124)	6.7±0.9 mmol/l	6.8±0.9 mmol/l	p = 0.41

LBW = low birth weight, IPTp = intermittent preventive treatment of malaria during pregnancy.

**Figure 1:**
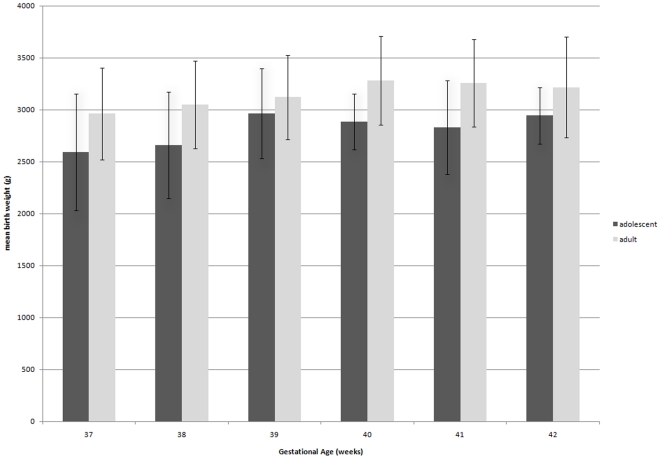
Birth weight of term-newborns born to adolescent and adult mothers for each week of gestation. Bars show mean birth weight and standard deviations for adolescent (≤16 years) and adult (>16 years) mothers.

As preterm delivery is the most important risk factor for low birth weight, a separate analysis of differences in birth weight between adolescent and adult mothers was carried out in the subset of term newborns (n = 535). Among these, the proportion of infants with low birth weight was 16% (6/37) in adolescents compared to 5% (25/498) in adults (OR: 3.7, 95%CI: 1.4–9.6). Accordingly, term newborns born to adolescent women had an average birth weight which was 315g lower than newborns born to adult women (2835±432g versus 3150±438g, p<0.0001, n = 535). Furthermore, mean birth weight was lower in newborns of adolescent than of adult mothers for each completed week of gestation (Figure 1).

### Multivariable logistic regression analysis of health outcomes

The impact of young maternal age on the probability of low birth weight was further analyzed in multivariable logistic regression analysis. The following dichotomized variables were used for logistic regression analysis with the occurrence of low birth weight as dependent variable: gestational age (preterm <37 vs. term ≥37 weeks of gestation), maternal age (≤16 or >16), parity status (nulliparity vs. multiparity), intake of intermittent preventive treatment of malaria during pregnancy (<2 vs. ≥2 doses), use of bednets (y/n) and *P. falciparum* infection at delivery (y/n).

The model showed an acceptable fit (chi2 = 58.9, df = 6, p<0.0001) and preterm delivery (adjusted OR 7.4 95% CI: 4.0–13.7, p<0.0001), young maternal age ≤16 (adjusted OR 2.9 95% CI: 1.5–5.6, p = 0.03) and parity (adjusted OR 1.9 95% CI 1.2–3.0, p = 0.01) showed a statistically significant influence on the probability to deliver an infant with low birth weight. Use of bednet, intake of intermittent preventive treatment of malaria, and *P. falciparum* infection at delivery had no significant impact on the probability of low birth weight in this model. Table 2 shows unadjusted and adjusted odds ratios for all variables used in the multivariable logistic regression model for the occurrence of low birth weight.

**Table 2:** Multivariable logistic regression analysis for delivery of an infant with low birth weight.

Category	Variable	N° of deliveries with LBW (%)	Univariable OR (95%CI)	Adjusted OR (95%CI) n = 529		Adjusted OR (95%CI) (including No. of consultations) n = 298	
**Gestational Age**	Preterm	38/126 (30.1)	7.0 (4.1–11.9)	**7.4 (4.0–13.7)**	**P<0.0001**	**9.5 (3.9–23.5)**	**P<0.0001**
	Term	31/535 (5.8)					
**Maternal Age**	≤16 years	13/57 (22.8)	2.9 (1.5–5.6)	**2.7 (1.1–6.5)**	**P = 0.03**	1.5 (0.3–8.2)	P = 0.65
	>16 years	67/718 (9.3)					
**Parity status**	Nulliparity	39/275 (14.2)	1.9 (1.2–3.0)	**2.3 (1.2–4.6)**	**P = 0.01**	1.0 (0.4–2.5)	P = 0.94
	Multiparity	39/481 (8.1)					
**Intake of IPTp**	<2 doses	36/278 (12.9)	1.6 (1.0–2.6)	1.4 (0.8–2.6)	P = 0.25	1.4 (0.6–3.7)	p = 0.46
	≥2 doses	36/415 (8.7)					
**Use of Bednet**	No	30/317 (9.5)	0.8 (0.5–1.3)	0.6 (0.3–1.2)	P = 0.13	0.7 (0.3–1.8)	P = 0.49
	Yes	40/331 (12.1)					
**Periph. Parasitaemia**	Positive	1/13 (7.7)	0.7 (0.1–5.6)	0.7 (0.1–7.1)	P = 0.7	2.01 (0.2–28.5)	P = 0.60
	Negative	79/762 (10.4)					
**No. of Consultations**	≤3 visits	22/125 (17.6)	3.3 (1.6–6.7)			**2.5 (1.0–6.3)**	**P<0.05**
	>3 visits	14/231 (6.1)					

LBW = low birth weight, OR = Odds Ratio, IPTp = intermittent preventive treatment of malaria during pregnancy.

### Impact of antenatal care visits on health outcome in adolescent and adult women

Information on the number of antenatal care visits was available only in a subset of mothers (n = 356) and a separate analysis was therefore performed. Patient characteristics (age, study site, birth weight, and gestational age) in this subset did not differ significantly from the main study population (data not shown).

Adolescent mothers had attended significantly less antenatal care visits than adult mothers (3.3±1.9 versus 4.4±1.9 mean visits, p<0.01, n = 356) and the difference in the frequency of consultations remained statistically significant after restriction to nulliparous women only (3.3±1.9 vs. 4.5±1.9 p<0.01, n = 133).

Multivariable regression analysis of the probability to deliver a low birth weight infant was performed in this subset of mothers using the same logistic regression model as for the main study population. In addition, the number of antenatal consultations (dichotomized as more than 3 vs. equal or less then 3) was entered as further variable into this analysis. This modified model again showed an acceptable fit (chi2 = 41.1, df = 7, p<0.0001), and prematurity (adjusted OR 9.5 95%CI 3.9–23.5, p<0.0001) and less than 3 antenatal care visits (adjusted OR 2.5a, 95%CI 1.0–6.3, p<0.05) were the only variables significantly associated with the occurrence of low birth weight (Table 2).

## Discussion

Due to conflicting results from previous epidemiologic studies and a lack of data from Sub-Saharan Africa, we aimed to analyze the risk of adolescent pregnant women to give birth to a low birth weight infant in the Central African country of Gabon. Our data indicate that the probability to deliver an infant with low birth weight was more than doubled for adolescent women compared to mothers older than 16 years. In multivariable analysis the probability of giving birth to a low birth weight infant remained significantly higher for adolescent than for adult mothers after correction for preterm delivery, parity and other established risk factors. Our data are in line with a previous report from neighboring Congo – a region with comparable prevalence of early pregnancy – showing an even higher proportion of low birth weight infants (37%) in young women [19]. These findings provide strong evidence that adolescent pregnancy constitutes an independent risk factor for low birth weight in Central Africa and underline the public health importance of adolescent pregnancies in this part of Africa.

Several reasons for the high risk of delivering a low birth weight infant by adolescent mothers have been discussed in scientific literature. Anatomic immaturity and continued maternal growth may represent biologic growth barriers for the fetus [10]. Moreover, adolescent mothers may represent a particularly disadvantaged risk group characterized by low socioeconomic status, financial income and level of education, or by an increased vulnerability to violence and sexual abuse [20]. Similarly, the socio-cultural perception of young maternal age may have an influence on health related behaviour of adolescent mothers. Since no systematic information on the aforementioned potential confounders was available for our study population the influence of these factors to the excess risk for low birth weight cannot be conclusively assessed.

Interestingly other well-established risk factors for low birth weight including the use of insecticide-treated bednets, adherence to intermittent preventive treatment of malaria in pregnancy, and infection with *P. falciparum* did not play a major role for adverse birth outcome in our study population. This finding may be explained by the low rate of *P. falciparum* infections in peripheral blood samples in our study population. Although placental sampling may have improved the detection of pregnancy associated malaria these results are in line with a recent epidemiologic survey on the effectiveness of intermittent preventive treatment of malaria in pregnancy in our study region [8]. In contrast, the number of antenatal care visits showed a statistically significant influence on the probability of delivering a low birth weight infant, and adolescent mothers had significantly less antenatal health care visits than adult mothers. Most importantly, no statistically significant difference in the risk of delivering a low birth weight infant was observed for adolescent mothers in multivariable analysis after correction for the number of antenatal care visits. These data indicate that the excess risk for low birth weight in young pregnant women may be preventable by optimized antenatal care. Future prospective studies evaluating targeted antenatal care programmes for adolescent mothers may help to definitely answer this hypothesis.

To better appreciate potential differences of intrauterine growth retardation during gestation and to better understand the interplay between preterm delivery and low birth weight, several sub-analyses have been performed including restriction of the study population to term deliveries and graphical depiction of birth weight for each week of gestation. All these analyses conclusively showed consistently lower birth weights for newborns of adolescent mothers compared to adults. This finding strongly indicates that low birth weight is not explained by the more frequent occurrence of preterm delivery in adolescent mothers and similarly that intrauterine growth retardation is a consistent phenomenon in adolescent pregnancies for all gestational ages.

Based on our data adolescent women constitute a special risk group of pregnant women. Since young pregnant women differ in many aspects from adults, routine antenatal health care programs may be insufficient for their needs. In a recent study from Zimbabwe, transport costs and costs for prenatal services have been characterized as major factors influencing adolescents' late or non-utilization of prenatal services [21]. In the same study the limited knowledge of young women about antenatal care programs and the fear of HIV testing have been further obstacles to efficient antenatal care. It may therefore seem desirable to establish dedicated health programs for adolescent women specifically addressing the needs and constraints of this special risk group.

The frequency of pregnancy during adolescence is highly variable in Africa [14]–[16]. Cultural and religious norms may be one of the reasons for these demographic differences. In addition, these cultural factors may modify health care seeking behavior of young pregnant women and may therefore constitute by themselves confounding factors for pregnancy outcome. Our analysis is restricted to the medical and epidemiologic aspects of adolescent pregnancies and is not intended to advise on the value of early pregnancy in itself as this appreciation is considered being part of the cultural identity of societies.

Possible limitations of our study include the fact that low birth weight – our primary outcome measure – is a surrogate marker for adverse pregnancy outcome. Although widely accepted and used as validated endpoint in clinical trials evaluating public health interventions in Africa, its significance as causal factor for disease has been discussed controversially [22]. However, birth weight remains an extremely powerful predictor of a newborn's chance for survival and is an easily assessable marker for evaluation of pregnancy outcomes and seems therefore still one of the most robust endpoints in epidemiologic studies of pregnancy.

The analysis focuses on women delivering live infants in health facilities and does therefore neither account for early abortions nor for home deliveries. Since home deliveries are estimated to occur in approximately 10% in our study area it is however not anticipated that this constitutes an important bias in this study [23]. A further limitation of our study is the limited information on the number of antenatal care visits, which was available only in a subset of participants in this study. Therefore definite conclusions about the importance of antenatal care are difficult to draw and further prospective studies with higher sample sizes are required. Finally, several other factors with known influence on pregnancy outcome including other infectious diseases (e.g. HIV, syphilis), anthropometric and socioeconomic characteristics of the mothers such as information on the educational level, marital status, and – in our study area less frequently encountered – exposure to tobacco and alcohol were not evaluated in this survey and could therefore not be included in multivariable analysis. The importance of these factors for the pathophysiology of low birth weight in the group of adolescent mothers can therefore not be answered conclusively by our study.

Our results convincingly demonstrate the importance of adolescent age and antenatal care for the improvement of pregnancy outcomes in Central Africa. Large prospective interventional studies are needed to further characterize adolescent pregnancy as risk factor for low birth weight, to evaluate potential strategies for the prevention of low birth weight in adolescent women, and to better appreciate the importance of adolescent pregnancy in different epidemiologic and demographic settings in Africa.
